# Neurophysiological correlates of mismatch in lexical access

**DOI:** 10.1186/1471-2202-6-64

**Published:** 2005-11-11

**Authors:** Claudia K Friedrich

**Affiliations:** 1 University of Konstanz, Department of Linguistics, Universitaetstrasse 10, P.O.Box D25, D-78457, Konstanz, Germany; 2 University of Hamburg, Department of Biological Psychology and Neuropsychology, Von-Melle-Park 11, D-20146 Hamburg, Germany

## Abstract

**Background:**

In the present study neurophysiological correlates related to mismatching information in lexical access were investigated with a fragment priming paradigm. Event-related brain potentials were recorded for written words following spoken word onsets that either matched (e.g., *kan *– *Kante *[Engl. edge]), partially mismatched (e.g., *kan *– *Konto *[Engl. account]), or were unrelated (e.g., *kan *– *Zunge *[Engl. tongue]). Previous psycholinguistic research postulated the activation of multiple words in the listeners' mental lexicon which compete for recognition. Accordingly, matching words were assumed to be strongly activated competitors, which inhibit less strongly activated partially mismatching words.

**Results:**

ERPs for matching and unrelated control words differed between 300 and 400 ms. Difference waves (unrelated control words – matching words) replicate a left-hemispheric P350 effect in this time window. Although smaller than for matching words, a P350 effect and behavioural facilitation was also found for partially mismatching words. Minimum norm solutions point to a left hemispheric centro-temporal source of the P350 effect in both conditions. The P350 is interpreted as a neurophysiological index for the activation of matching words in the listeners' mental lexicon. In contrast to the P350 and the behavioural responses, a brain potential ranging between 350 and 500 ms (N400) was found to be equally reduced for matching and partially mismatching words as compared to unrelated control words. This latter effect might be related to strategic mechanisms in the priming situation.

**Conclusion:**

A left-hemispheric neuronal network engaged in lexical access appears to be gradually activated by matching and partially mismatching words. Results suggest that neural processing of matching words does not inhibit processing of partially mismatching words during early stages of lexical identification. Furthermore, the present results indicate that neurophysiological correlates observed in fragment priming reflect different aspects of target processing that are cumulated in behavioural responses. Particularly the left-hemispheric P350 difference potential appears to be closely related to fine-grained activation differences of modality-independent representations in the listeners' mental lexicon. This neurophysiological index might guide future studies aimed at investigating neural aspects of lexical access.

## Background

Spoken word comprehension requires a listener to identify a single word among thousands of representations stored in her mental lexicon. Behavioral research suggests that the incoming auditory signal activates multiple lexical representations that match the signal [1], but also representations with partial mismatch to the input [2,3]. Pseudowords like *gabinet *or *mabinet*, for example, have been shown to activate the word *cabinet*. However, this activation is decreased as compared to a complete match. Thus, the matching of input and stored representations results in a specific activation pattern. Words that completely match the input are strongly activated. Words that partially mismatch the input are less strongly activated. Competition among activated candidate words has been postulated as a mechanism that reduces the number of activated words [4,5]. Strongly activated words inhibit less strongly activated words.

Both, graded lexical activation and competition, have been previously investigated using word fragment priming. A word fragment, which is commonly the onset of a spoken word, is immediately followed by a visual word or a meaningless letter string (pseudoword). Participants are asked to decide whether they saw a word or not. Faster responses for words that match the fragment as compared to unrelated control words have been found. For example, responses to *music *are faster when it follows *mus *than when it follows *viba*. This facilitation has been interpreted as reflecting the activation that modality-independent representations of matching words receive from the fragments [6,7] (see [8] for an introduction into from priming paradigms).

Different amounts of overlap between fragment and word modulate reaction times in fragment priming. For example, responses are faster when a fragment has the same stress as the target word than when fragment and target word differ in stress [9,10]. A word like *music*, with stress on the first syllable, is responded to faster when it is preceded by stressed *mus *than when it is preceded by unstressed *mus*. This result illustrates graded lexical activation depending on goodness-of-fit between fragment and word. Furthermore, inhibition has been found when fragments were taken from competitor words. Responses are faster when a word is preceded by an unrelated fragment than when the same word is preceded by a partially mismatching fragment for which a better completion exists [11]. For example, *abon *taken from the Spanish word *abonado *(Engl. subscriber) inhibits processing of *abanico *(Engl. fan).

Event-related brain potentials (ERPs) recorded for targets in fragment priming reveal a previously undescribed left-hemispheric brain potential [12]. It is called the P350 and differentiates matching words from unrelated control words. Difference waves resulting from the subtraction of matching words from unrelated control words reveal more positive amplitudes with a maximum peak at 350 ms. Comparable P350 difference waves have been shown for visual fragments preceding visual words. Therefore, it was concluded that the P350 reflects neural processing related to the identification of modality-independent lexical representations. Furthermore, subtle differences in the speech signal such as pitch contour modulate the P350 effect. Pitch contour is an important parameter that marks lexical stress. P350 effects reveal that words with a stress pattern that matches the pitch contour of the input are stronger activated than words that do not match in their stress pattern [13].

Besides the P350 effect, enhanced N400 amplitudes are reliably found for unrelated control words in fragment priming. The N400 is a frequently observed ERP component in different language related tasks (see [14] for a review). It has been argued that the N400 amplitude does not reflect automatic lexical activation in priming tasks [15-17]. It appears more plausible from a vast amount of N400 results that the amplitude of this component is inversely related to the effort needed to integrate an incoming word in a preceding context formed by a sentence or a single priming stimulus. Accordingly, the N400 might rather be related to post-lexical matching and integration processes than to lexical activation in fragment priming [12]. A lexical account to the N400 in word fragment priming is also challenged by the fact that its amplitude is not sensitive to fine-grained activation differences as a function of pitch [13]. Therefore, we interpreted the N400 as a correlate of strategic effects that are assumed to assist lexical decisions in a priming situation [18-20]. Possible mechanisms that speed up yes-responses to matching words might be a rough phonological matching between prime and target or the expectation of a phonological form, which is established by the fragment. A phonological account to the N400 is supported by N400 reduction found for rhyming words [21-23] or word stem priming [24,25].

The present study aimed to explore ERP correlates of the activation of partially mismatching words following fragments for which better completions exist. Monosyllabic word onset fragments were extracted from German word pairs that had identical first syllables except of the vowel (e.g., *Kante *[Engl. Edge] and *Konto *[Engl. account]). Three types of fragment-word pairs summarized in Table 1 were tested: In a *Match *condition, words were preceded by completely matching fragments. In a *Mismatch *condition, the same words were preceded by fragments extracted from their pair members. In *Control *conditions, fragments were followed by unrelated control words. Neural correlates of graded lexical activation are shown. However, neither neurophysiological data nor behavioral results reveal inhibition of partially mismatching words during this early phase of lexical identification.

## Results and discussion

### Reaction times and error rates

**Figure 1:**
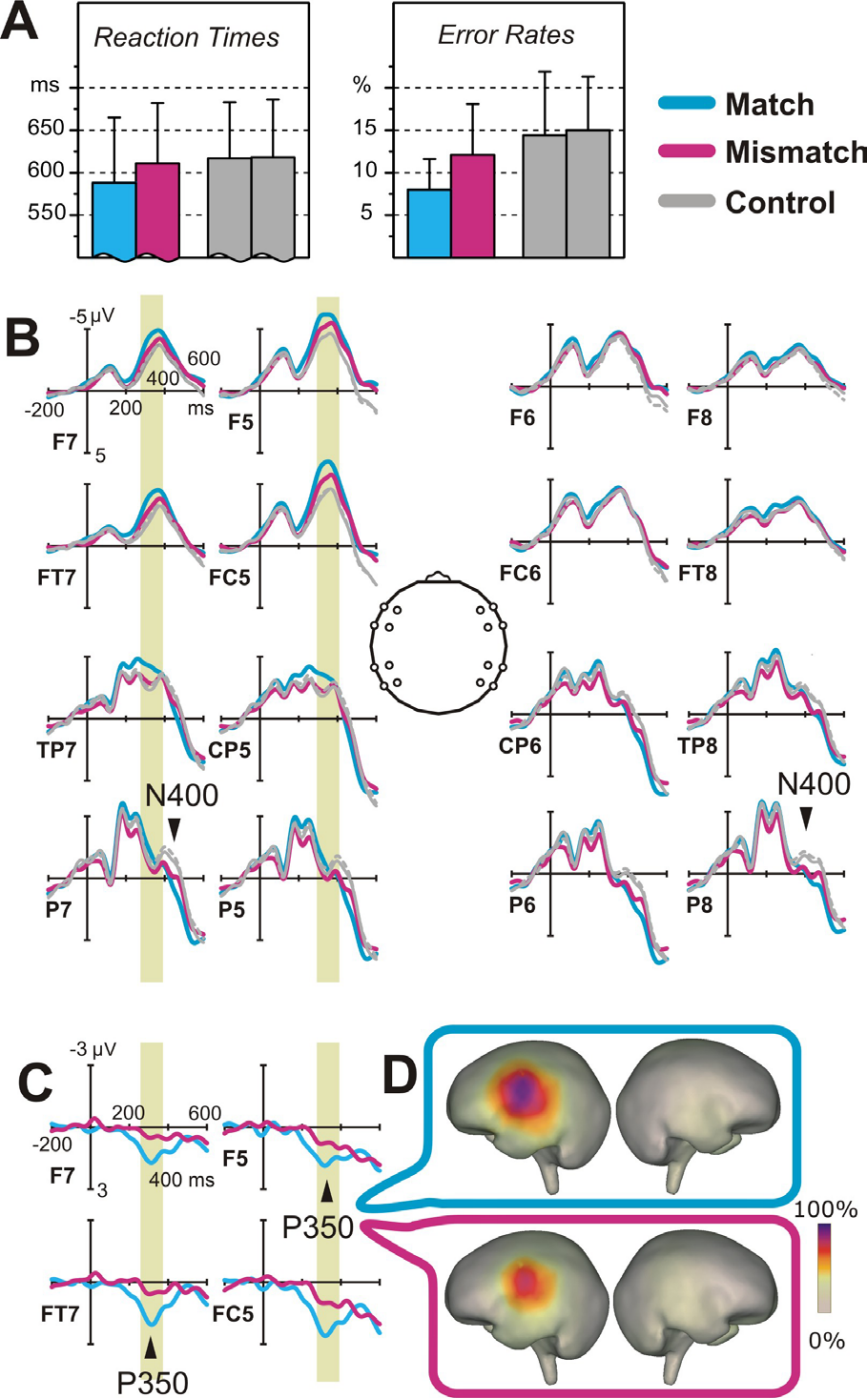
A) The first part of the figure shows reaction times in milliseconds and error rates in percent for words preceded by completely matching fragments (light blue), for the same words preceded by partially mismatching fragments (pink), and for unrelated control words (grey). Note that error bars represent standard deviations. B) Grand mean averages representing ERPs for 16 selected electrode places over the right and left hemisphere are plotted in this part of the figure (matching words in light blue; mismatching words in pink; unrelated control words in grey solid and dotted lines). Electrode positions are illustrated in the map. The time window for which statistical analyses of the P350 effect were conducted is highlighted in grey. C) Difference waves for four selected left-hemispheric electrode places representing the P350 effect are shown in this part of the figure (unrelated control words – matching words in light blue; unrelated control words – mismatching words in pink). Again the time window of statistical analysis of the P350 effect is highlighted in grey. D) This part of the figure shows minimum norm solutions estimating the neural sources underlying the P350 difference waves for matching words (above) and for mismatching words (below). Colors represent strength of dipole activation in percent relative to the highest level of activation in the match condition at the P350 peak (352 ms).

Mean reaction times and error rates are shown in Figure 1A. Behavioural measures were subjected to one-way ANOVAs with the three-level factor *Relatedness *(matching words vs. partially mismatching words vs. unrelated control words). A main effect of *Relatedness *allowed further step-down analyses of differences between conditions, F(2,46) = 41.84, Greenhouse-Geisser epsilon = 0.90, corrected p < .001. Responses to completely matching words were faster than responses to unrelated control words, t(1,23) = 58.81, p < .001. Similarly, responses to partially mismatching words were faster than responses to unrelated control words, t(1,23) = 6.04, p = .02. Nevertheless, subjects' responses were faster for matching than for partially mismatching words, t(1,23) = 46.01, p < .001. The same pattern of results was observed for error rates for which again a main effect of *Relatedness *was observed, F(2,46) = 17.10, Greenhouse-Geisser epsilon = 0.76, corrected p < .001. Subjects made less errors for matching words than for unrelated control words, t(1,23) = 21.82, p < .001. Similarly, subjects made less errors for partially mismatching words than for unrelated control words, t(1,23) = 7.89, p < .01. Nevertheless, responses to matching words were more accurate than responses to partially mismatching words, t(1,23) = 14.90, p < .001.

In sum, reactions were faster and more accurate for matching words than for partially mismatching words. This finding goes in line with behavioral results of previous fragment priming experiments [6,7,9-11]. However, the fact that responses for partially mismatching words were not slower than responses for unrelated control words does not replicate previous work. Remember that in a former study subjects responded faster to unrelated control words (e.g., *indi *– *abanico*) than to partially mismatching words (e.g., *abon *– *abanico*). This has been taken as evidence that better matching completions inhibit partially mismatching words [11]. The present results indicate that inhibition of partially mismatching words is not an obligatory finding in word fragment priming.

Taken together present and previous work, it might be concluded that the lengths of the fragments is a factor that modulates inhibition effects. In contrast to the former study, in which disyllabic fragments were presented as primes [11], monosyllabic fragments were used in the current experiment. Accordingly, the present results suggest that effective competition needs (i) information exceeding the first syllable of a spoken word and/or (ii) time to exert inhibitory effects. With respect to (i): Single syllables as used in the present experiment might fully and partly activate a large number of competitors that do not effectively inhibit each other. In contrast, disyllabic fragments as used in the previous study might fully activate only a few or at least only one word, which effectively inhibits partly activated words. With respect to (ii): Additional processing time provided by disyllabic fragments, which have a longer duration than monosyllabic fragments, might stabilize inhibitory effects. Further research has to explore both possible influences on competition effects.

### ERPs

Mean amplitudes for matching words, partially mismatching words and unrelated control words are shown in Figure 1B for eight selected electrode sites, respectively. Waveforms were characterized by an N1-P2 complex followed by negativity between 200 and 400 ms, most prominent over frontal electrode positions. Amplitudes of left hemispheric electrodes were sensitive to the experimental manipulation in the time range between 300 and 400 ms. Difference waves showed characteristic P350 effects (see Figure 1C). Analysis of P350 effects was identical to a former study with syllabic fragments [13]. Starting at approximately 350 ms, an N400 component was observed over bilateral posterior electrode positions. The N400 effect began earlier and was of shorter duration than that observed in previous fragment priming studies [12,13]. It was examined using a time window between 350 and 500 ms.

### P350: 300 to 400 ms

A three-way ANOVA with factors *Relatedness *(matching words vs. partially mismatching words vs. unrelated control words), *Hemisphere *(left vs. right electrode positions), and *Region *(anterior vs. posterior electrode positions) was applied to analyze P350 effects. This analysis yielded significant interactions of the factors *Relatedness*, and *Hemisphere*, F(2,46) = 16.07, Greenhouse-Geisser epsilon = 0.98, corrected p < .001; and *Relatedness, Region *and *Hemisphere*, F(2,46) = 10.78, Greenhouse-Geisser epsilon = 0.75, corrected p = .01. Amplitudes for matching words differed from unrelated control words over both left hemispheric ROIs, both t(1,23) ≥ 5.26, both p ≤ .03. This suggests lexical activation of matching words. Partially mismatching words also showed some degree of lexical activation. Their amplitudes differed from amplitudes of control words over the left anterior ROI, t(1,23) = 5.92, p = .02. However, the fact that amplitudes for matching words differed from partially mismatching words over both left hemispheric ROIs indicates strongest activation for matching words, both t(1,23) ≥ 11.92, both p ≤ .002.

To sum up, ERPs elicited over the left hemisphere were sensitive to the experimental manipulation in the time window of the previously reported P350 deflection [12,13]. Over left-temporal scalp regions, a positive-going effect with enhanced amplitudes for unrelated control words as compared to matching words replicates former P350 results (see electrodes TP7, CP5, P7, P5 in Figure 1B). In contrast, negative-going ERPs with enhanced amplitudes for matching words were observed in the time window of the P350 over left-anterior regions (see electrodes F7, F5, FT7, FC5 in Figure 1B). What remains stable across studies is that subtraction of ERPs for matching or partially mismatching words from ERPs for unrelated control words results in positive-going difference waves with a maximum at 350 ms. Therefore, it appears more appropriate to apply the label 'P350 effect' to these difference waves than to a positive-going deflection in the ERPs.

P350 effects appear to differ with respect to their scalp topography on an anterior-to-posterior dimension over the left hemisphere. In the present study P350 effects were pronounced over temporo-frontal electrode positions. In contrast, only temporal electrodes were involved in P350 effects found in previous studies [12,13]. An obvious cause for different P350 topographies might be the earlier onset of the N400 effect in the present study as compared to the previous studies. The N400 also shows a posterior scalp distribution, but counteracts the P350 effect in polarity of the elicited differences. The earlier beginning of the N400 effect in the present study may have canceled out the posterior part of the P350 effect resulting in the observed temporo-frontal scalp topography.

The present results are consistent with the idea that P350 effects are closely correlated to the activation status of lexical entries in a modality-independent mental lexicon. Matching words, which are strongly activated by the input, elicit large P350 difference waves. Partially mismatching words, which are less strongly activated by the input, elicit reduced P350 difference waves. However, from the present and the previous results it is difficult to distinguish whether the P350 effect reflects a surplus in positivity for unrelated control words or a surplus in negativity for matching words. As already discussed P350 effects were related to different ERP deflections across the present study and previous studies. It has been suggested that reduced positive-going amplitudes for matching words are a correlate of facilitated lexical identification resulting from pre-activation of lexical entries [12,13]. In contrast, enhanced negative ERP amplitudes for matching words over left temporo-frontal electrodes, which were observed in the present study, might suggest that P350 effects directly result from the activation status of lexical entries.

Negative-going left temporo-frontal ERPs in the time window of the P350 effect look very similar to an N330 effect reported earlier [26]. The N330, which also shows a temporo-frontal scalp distribution, was found to be enhanced for words primed by semantic associates as compared to unprimed words. This contrasts the N400 priming effect classically showing reduced amplitudes for words with semantic relation to their preceding primes (see [14]). Both the N330 effect in semantic priming and the P350 effect in word fragment priming can be functionally separated from the N400 effect. Interestingly enough, the N330 was interpreted as a correlate of semantic or lexical activation. This is in line with the present interpretation of the P350 difference wave being related to lexical activation. It remains to be investigated whether the neural activation mechanism that is reflected in the P350 difference wave might also underlie the previously reported N330.

Minimum norm calculations were conducted to elucidate the localization of neural processes underlying P350 difference waves. Results suggest a left centro-temporal origin of neural sources engaged in the processing of both matching and partially mismatching words (see Figure 1D). Crucially, minimum norm solutions in both conditions differ only in strength of dipole activation but not in estimated neural sources. These results clearly point to a unique underlying left-temporal source for processes that are involved in the mapping of speech input onto modality-independent lexical representations. The underlying neuronal network was stronger activated by matching than by partially mismatching words. The minimum norm solutions suggest a relation between the P350 effect in the ERP and an M350 effect found in magnetic brain responses [27]. The M350 has been characterized as an automatic early component, which appears to be related to lexical access in visual word processing [28]. Time ranges of P350 difference waves and M350 are comparable [12]. Furthermore, source analyses for both neurophysiological correlates point to left-temporal neural activation. Both, the P350 and the M350, might index automatic spreading activation across lexical entries. They provide new means to explore aspects of early word processing and the underlying neuronal mechanisms.

### N400: 350 to 500 ms

N400 effects were analyzed using the same statistical design as for P350 effects (see previous section). The three-way ANOVA yielded significant interactions of the factors *Relatedness *and *Region*, F(2,46) = 31.86, Greenhouse Geisser epsilon = 0.69, corrected p < .001, *Relatedness *and *Hemisphere*, F(2,46) = 5.52, Greenhouse Geisser epsilon = 0.88, corrected p < .01, and *Relatedness*, *Region*, and *Hemisphere *F(2,46) = 9.21, Greenhouse Geisser epsilon = 0.68, corrected p < .01. For both posterior ROIS matching words elicited reduced amplitudes of the N400 as compared to unrelated control words, both t(1,23) = 6.18, both p = .02. Similarly, partially mismatching words elicited reduced amplitudes of the N400 over both posterior ROIs, both t(1,23) ≥ 5.89, both p ≤ .03. However, N400 amplitudes for matching and partially mismatching words did not differ significantly, t(1,23) ≤ 1.52, n.s. Thus, in contrast to P350 difference waves and reaction times, N400 amplitude did not differentiate matching words and partially mismatching words.

The present N400 results go in parallel with the earlier finding that the N400 is not sensitive to a mismatch between the pitch of a fragment and the stress pattern of a succeeding word [13]. Taken together, results of both studies indicate an insensitivity of the N400 to subtle differences between fragment and word. They support an interpretation of the N400 as a correlate of neural processes that operate at a post-lexical level, and indicate that behavioral responses in fragment priming are modulated by more than lexical activation. Phonological matching has been postulated as a possible mechanism that is reflected in the N400 in fragment priming [13]. Note that both, matching words and partially mismatching words, are phonologically related to the fragments (e.g., kon-KONTO or kan-KONTO), whereas unrelated control words show no phonological relation to their primes (e.g., kon-SALTO). The fact that both, matching words and partially mismatching words, elicit reduced N400 amplitudes confirms the assumption that processes underling the N400 provide a superficial matching or expectation that speeds up yes-responses to words with some phonological relation to the fragment. A phonological account to the N400 is also suggested by N400 reduction for rhyming words [21-23] or for word stem priming [24,25].

## Conclusion

The present results indicate that fragmentary word information modulates different aspects of neural target processing. This is reflected by two separate ERP correlates, namely P350 and N400 effects. Both can be distinguished as separate neurophysiological deflections in accordance to several relevant characteristics (see for example [29]). They differ with respect to latency, scalp topography, polarity of the elicited differences, and sensitivity to the experimental manipulation. Therefore, P350 and N400 effects probably reflect at least two different neuronal processes, which both precede and modulate behavioral responses in fragment priming. P350 effects on the one hand appear to be related to the fine grained mapping of the acoustic input onto lexical representations. N400 effects on the other hand might be related to phonological matching between fragment and word. This argumentation leads to the conclusion that behavioral data, which are preceded by P350 and N400 effects, reflect outcomes of both processes. ERPs, in particular P350 effects, allow investigating lexical activation more directly than behavioral data.

Both, reaction times and P350 difference waves, suggest that partially mismatching lexical representations, which diverge from spoken word fragments only in the vowel of the first syllable, receive slight activation from the speech input. In contrast to earlier findings [11], both measures reveal that partially mismatching representations are not inhibited by better matching completions. Thus, the present data do not support a strong notion of competition between activated lexical candidates at early stages of lexical processing. They reveal that the human speech recognition system does not strictly inhibit alternative candidates from early lexical activation. Factors that modulate competition effects, such as the length of the fragments, have to be focus of future research.

Finally, the neuronal sources underlying the activation of modality-independent lexical representations can be studied with fragment priming in more detail. ERPs in word fragment priming indicate that activation of representations that receive input from both, spoken and written words, appears to be a left-hemispheric function with underlying neuronal sources in the centro-temporal cortex. Although these neuronal sources appear to be separate from written word from representations in the left fusiform gyrus [30], a relation to speech representations in the left superior temporal sulcus [31] can be assumed. In light of the primacy of auditory language comprehension in ontogenetic development, the question emerges whether modality-independent lexical representations are identical to speech representations. The P350 effect seems to be a powerful neurophysiological means to address this and related questions regarding neuronal bases of lexical access in human language comprehension.

## Methods

### Participants

Twenty-four right-handed volunteers (12 females, 12 males, mean age 22 years) from the Leipzig Max Planck Institute of Human Cognitive and Brain sciences subject pool took part in the experiment. All subjects were right-handed native speakers of German with no reported hearing or neurological problems and normal or corrected-to-normal vision.

### Stimuli and procedures

Sixty word pairs served as carrier for prime fragments. Words in a word pair shared the same first syllable with exception of the vowel (e.g. *Kan.te *and *Kon.to*, dots indicate the end of the extracted fragment). To control for co-articulation, the phoneme following the fragment was identical for words in a word pair. All carrier words were spoken by a female native speaker of German and recorded using an analog recorder. Items were then digitized at a sampling rate of 44 kHz with 16-bit analog-to-digital conversion on a PC computer using the software *Computerized Speech Lab *(CSL, ^® ^Kay Elemetric Corp.). Fragments were created from the digitized signals with *CoolEdit *(^® ^Syntrillum Software Corp.). All fragments were taken from initially stressed words.

Written forms of the carrier words were presented as target words. Matching words were preceded by the prime extracted from their spoken form (e.g., *kon *– *Konto*, 120 trials). The same words also served as partially mismatching words. In the latter case they were preceded by fragments extracted from the respective pair member (e.g., *kan *– *Konto*, 120 trials). Control words were completely unrelated to the fragments in word onset (e.g.,*kon *– *Salto*, 240 trials). Unrelated control words were matched to the related words with respect to stress, number of syllables and number of letters, as well as with respect to word frequency, according to an online data base of German [32]. For example, *Salto *served as control word for *Konto*, whereas *Zunge *served as control word for *Kante*. In order to equalize the number of repetitions and to balance related and unrelated trials, unrelated control words were presented twice: Once in combination with the fragment from the matched target (*kon-Zunge*), and once in combination with the fragment taken from the targets' pair member (*kan-Zunge*).

In half of the trials pseudowords were presented. Pseudowords were created by interchanging the last one or two letters of two related words or two unrelated words. Pseudoword creation followed phonotactic rules of German. Pseudowords were combined with fragments in the same way as words: They matched the fragment (e.g., *kon *– *Konta*, 120 trials), mismatched in the vowel (e.g., *kan *– *Konta*, 120 trials) or were unrelated to the fragment (e.g., *kon *– *Salte*, 240 trials). The first presentation of targets was counterbalanced for the match, mismatch, and unrelated control conditions, as well as for the words and pseudowords.

Participants were comfortably seated in an electrically and acoustically shielded chamber. Visual stimuli were presented on a computer screen in front of the subjects. An experimental trial began with the presentation of a fixation cross at the centre of the screen. Participants were instructed to fixate this cross whenever it appeared. While the fixation cross remained on the screen, a spoken word onset fragment was presented via loudspeakers after 300 ms. Fragments had a mean duration of 321 ms (SD = 61). Loudspeakers were placed at the left and the right side of the screen. The fixation cross was replaced by a visual word or pseudoword in immediate succession to the auditory fragment. Visual stimuli were presented for 200 ms in uppercase white letters on a black background. The task was to judge as quickly and as correctly as possible whether or not the target was a word. Half the subjects made yes-responses via button press with the thumb of their left hand and no-responses via button press with the thumb of their right hand, for the remaining subjects response hands were reversed. Reaction times were measured from stimulus onset with a time-out of 1500 ms. The next trial started after a fixed inter-trial interval of 1000 ms after the behavioral response, or 2500 ms after onset of the visual stimulus if no response was given.

### Electrophysiological recording and data analysis

The EEG was recorded continuously (250Hz/22 bit sampling rate; DC amplifier by Twente Medical Systems) from 58 Ag-AgCl electrodes mounted in an elastic cap (Electro Cap International, Inc.) according to the international 10–10 system. All electrodes were referenced against the nose tip. Impedances were kept below 5 kO. Four further electrodes provided biploar recordings of the horizontal and vertical electro-occulogram (EOG). The vertical EOG was recorded from electrodes placed above and below the right eye; the horizontal EOG was recorded from electrodes at the outer canthus of each eye. An electrode placed at the sternum served as ground. Artifacts caused by facial and eye movements were rejected off-line when one of the EOG recordings exceeded a voltage change of 30 μV or more within 200 ms. Furthermore, a visual inspection of the raw EEG was carried out to eliminate drifts. ERPs were computed for the words starting from the beginning of the visual presentation up to 600 ms and with a 200 ms prestimulus baseline. Only correctly answered, artifact-free trials were included in the averaging procedure. For illustrative purposes only, the grand-average ERPs were smoothed off-line using a 10-Hz lowpass filter.

Minimum norm estimations were calculated using the *Source Analysis *module of BESA (^® ^MEGIS Software GmbH [33]). Grand average difference waves for the match and the mismatch condition were subjected to the *Source analysis *module as a whole segment. No additional filtering was applied to the source analysis. Depth weighting and spatio-temporal weighting (Dale & Sereno [34]) were used to calculate source solutions. Noise was estimated using the baseline of the match condition.

Responses shorter than 200 ms, and longer than 1500 ms were removed from both behavioral and ERP analyses. Percent of erroneous responses and reaction times calculated from the onset of the visual words to the subjects' responses were subjected to a one-way ANOVA with the three-level factor *Relatedness *(matching words vs. partially mismatching words vs. unrelated control words). Degrees of freedom for the three-level factor *Relatedness *were adjusted by using the Greenhouse-Geisser epsilon. Two additional factors served for analysis of ERP effects. To analyze lateral electrodes, the factor *Hemisphere *(left vs. right electrode positions) was included. To analyze anterior vs. posterior effects the factor *Region *(anterior vs. posterior electrode sites) was included. This resulted in four ROIs including 11 electrodes each (anterior left: F9, F7, F5, F3, FT9, FT7, FC5, FC3, T7, C5, C3; anterior right: F10, F8, F6, F4, FT10, FT8, FC6, FC4, T8, C6, C4; posterior left: TP9, TP7, CP5, CP3, P9, P7, P5, P3, PO7, PO3, O1; posterior right: TP10, TP8, CP6, CP4, P10, P8, P6, P4, PO8, PO4, O2). In case of significant interactions, t-tests were computed to evaluate differences among conditions. Only main effects of the factor *Relatedness *and interactions including this factor and leading to significant post-hoc comparisons are reported. Note, that the time windows and the ROIs applied to analyze the present ERP data were identical to that used in a former fragment priming study with syllabic fragments [13].

## Figures and Tables

**Table 1:** This table displays the experimental design of the present study, and gives examples of trials that were realized in the conditions. The same words were presented as related words, once in combination with a matching fragment, one in combination with a partially mismatching fragment. Similarly, unrelated words were presented twice to control for responses biases and repetition effects. Both types of control words are shown in the figures in order to illustrate that there were virtually no differences between both conditions. Statistical analyses included only one group of control words.

***Related words***	
**Match**	**Mismatch**

*kon-KONTO*	*kan-KONTO*
*kan-KANTE*	*kon-KANTE*
Σ = 120 trials	Σ = 120 trials

***Unrelated control words***	

*kon-SALTO*	*kan-SALTO*
*kan-ZUNGE*	*kon-ZUNGE*
Σ = 120 trials	Σ = 120 trials
